# Lignin from Micro- to Nanosize: Production Methods

**DOI:** 10.3390/ijms18061244

**Published:** 2017-06-10

**Authors:** Stefan Beisl, Angela Miltner, Anton Friedl

**Affiliations:** Institute of Chemical, Environmental and Biological Engineering, TU Wien, 1060 Vienna, Austria; angela.miltner@tuwien.ac.at (A.M.); anton.friedl@tuwien.ac.at (A.F.)

**Keywords:** lignin, nanoparticles, microparticles, precipitation, biorefinery

## Abstract

Lignin is the second most abundant biopolymer after cellulose. It has long been obtained as a by-product of cellulose production in pulp and paper production, but had rather low added-value applications. A changing paper market and the emergence of biorefinery projects should generate vast amounts of lignin with the potential of value addition. Nanomaterials offer unique properties and the preparation of lignin nanoparticles and other nanostructures has therefore gained interest as a promising technique to obtain value-added lignin products. Due to lignin’s high structural and chemical heterogeneity, methods must be adapted to these different types. This review focuses on the ability of different formation methods to cope with the huge variety of lignin types and points out which particle characteristics can be achieved by which method. The current research’s main focus is on pH and solvent-shifting methods where the latter can yield solid and hollow particles. Solvent shifting also showed the capability to cope with different lignin types and solvents and antisolvents, respectively. However, process conditions have to be adapted to every type of lignin and reduction of solvent demand or the integration in a biorefinery process chain must be focused.

## 1. Introduction

Many petrochemicals are produced from conventional crude oil-fed refineries, whereas it is anticipated that in future, many products and chemicals will be produced from biorefineries fed with lignocellulosic biomass such as agricultural residuals [1]. This renders the term ‘‘waste”, in the context of biomass processing terminology, obsolete as each production stream has the potential to be converted into a by-product or energy rather than waste [2].

However, lignin as the second most abundant biopolymer on earth after cellulose is underutilized in first-generation cellulosic projects. Only around 40% of the generated lignin is needed to cover the internal energy demand of a biorefinery [3,4]. However, its non-uniform chemical structure and limited industrial supply impedes the current industrial use of lignin despite various potential applications [5,6,7,8,9]. Nevertheless, the expected increase in available technical lignin from pulp and paper processes and lignocellulosic biorefineries as well as the unique chemical structure of lignin compels researchers to develop new value-added products [5,7]. Considering the development of the second generation biofuel production in the US alone, 62 million tons of lignin will be produced by 2022 [10].

Lignin is a highly irregularly branched polyphenolic polyether, consisting of the primary monolignols, p-coumaryl alcohol, coniferyl alcohol and sinapyl alcohol, which are connected via aromatic and aliphatic ether bonds [11] as well as non-aromatic C–C bonds. Lignins from different sources have varying shares of these monolignols. Roughly three different types of lignins can be distinguished: softwood lignins are comprised almost solely of coniferyl alcohol, hardwood lignins of both coniferyl and sinapyl alcohol and grass lignins of all three types [12].

In addition to the complexity of the biomass lignin structure given by the lignin, in many cases, currently applied pretreatment technologies increase the intricacy and inhomogeneity even further [13]. This adds additional challenges for lignin’s downstream processing and valorization [14]. The lignins from different processes can be roughly classified in Kraft lignin (KL), Soda lignin, lignosulphonates (LS), Organosolv lignins (OS), Steam-explosion lignin and enzymatic hydrolysis lignin (EHL) [5]. Each of the lignin classes is associated with particular changes in the chemical structure and properties [12]. However, lignin possesses many unique properties such as resistance to decay and biological attacks, UV absorbance, high stiffness, and antioxidation. Therefore, it has the potential as large-volume feedstock to produce high value products [6,15].

Most lignins are only soluble in water at alkaline pH, which is a major limitation for its application at an industrial scale but the potential for preparing aqueous lignin nanoparticle dispersions has been shown [16,17]. Therefore, the preparation of lignin nanoparticles and other nanostructures has gained interest among researchers during the last years. Nanostructured materials, especially in the 1–100 nm range, offer unique properties due to their increasing surface area [18], while their important chemical and physical interactions are governed by surface properties. Hence, a nanostructured material can have considerably different properties from a larger-dimensional material of the same composition [19]. Therefore, possible applications are given in alternative to toxic nanoparticles [16], for drug delivery systems [17], for delivery of hydrophobic molecules [20], and for enhancing the UV barrier, antibacterial [21], antioxidant properties [22] and reinforcement of polymers [23] or as electrode material [24].

In general, nanomaterials are classified by their geometries into particle, layered, and fibrous materials [25]. Several methods are capable of producing these different geometries. However, the above-mentioned difficulties of lignin’s heterogeneity also apply in the production of nano- and microstructured materials. Although some authors have acknowledged this, they could not provide a comprehensive overview of the topic [26,27,28,29]. Therefore, this review is dedicated to show the applied production methods used and how these methods can deal with different raw materials and control the final product. The focus is on nanomaterials composed largely of lignin and due to a somewhat vague definition of NPs [30], a dimensional range from a few nanometers to a few micrometers was chosen.

## 2. Production of Nano-/Microsized Lignin Materials

Nanoparticles can be formulated in different ways. Wurm et al. [26] divide them into three classes where: (1) the monomer is polymerized during the preparation process to eventually form nanostructures; (2) an insoluble polymer is subjected to a physical process resulting in nanoparticles; (3) a soluble polymer is cross-linked in a suitable way.

**Figure 1:**
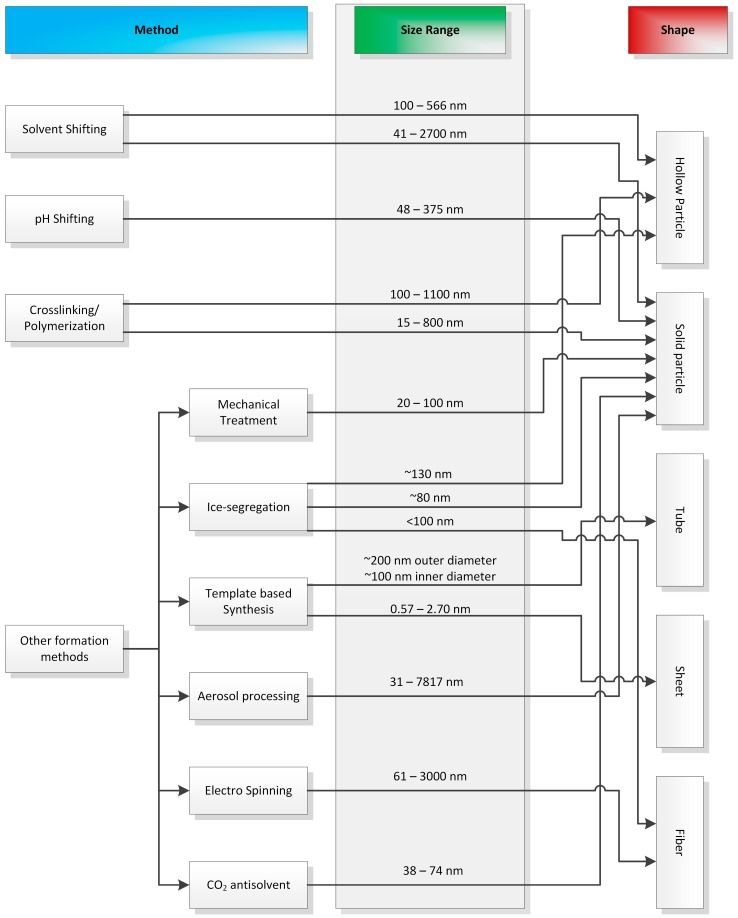
Illustration of the classes in which methods found in literature for nano- and micromaterial synthesis were divided into. The size range represents the sizes of the successfully synthesized structures. Resulting nano- and micromaterials were divided in five shape classes.

However, findings in the literature research led to a further division of these three classes, as well as structuring nanoparticles by the production method used and subsequently by the shape of the product as seen in Figure 1. The “size range” row shows the particle sizes or fiber/tube diameters, which could be successfully produced with the corresponding method. The general shape classes were defined as “Hollow Particle”, “Solid Particle”, “Tube”, “Sheet” and “Fiber”. The “Solvent Shifting”, “Cross-linking/Polymerization” and “Ice-segregation” method classes visibly resulted in more than one particular shape. Significantly, only changing the process parameters in the studied methods of classes “Solvent Shifting” and “Ice-segregation” led to either hollow or solid particles.

### 2.1. Solvent Shifting

Solvent shifting includes a solution of an organic compound in a water miscible organic solvent, which is mixed with an excess of water, and nanoparticles are generated due to their decreasing solubility [26]. The solvent shifting process has a wide range of potential applications and presents a straightforward system. However, the major limitation of this process is the low solids content (around 1 wt %), which can be obtained [31,32,33].

The obtained precipitates showed either a solid or a hollow spherical-like shape. Therefore, the methods were subdivided depending on their final shapes.

#### 2.1.1. Solid Particles

Several methods and process conditions were found, leading to solid spherical-like particles. All methods mentioned use water as antisolvent whereas a variety of different solvents is used, namely tetrahydrofuran (THF), acetone/water and dimethyl sulfoxide (DMSO).

Lievonen et al. [34] dissolved softwood lignin from a Kraft process (LignoBoost) in THF at various concentrations from 0.1 to 10 g/L. For the precipitation step, the solution was placed into a dialysis bag (6–8 kDa) and immersed in excess of deionized water for at least 24 h. This process resulted in spherical colloidal lignin nanoparticles with an average diameter between 200 and 500 nm, with a minimum average diameter at an initial lignin concentration of 1 g/L. The nanolignin dispersions were very stable in pure water because no specific aggregation occurred within 60 days, which was explained by the relatively high negative ζ-potential of about −60 mV in pure water at pH values from around 5 to 11. This work yielded more spherical nanoparticles compared to other methods [16,35,36], which represents an important advantage for advanced applications. Particles could be dried and dispersed using sonication. Furthermore, adsorption of cationic poly (diallyldimethylammonium chloride) (PDAC) on the particle surface could be changed from negative to positive values. Notably, a nearly-identical procedure using ethylene glycol (EG) and KL (Sigma-Aldrich) instead of THF and softwood KL did not result in uniform, round shapes, but in rather irregular ones.

Figueiredo et al. [37] used the same method as described above including identical lignin [34]. The dynamic light scattering results revealed an average hydrodynamic radius of 221 ± 10 nm, which is in good agreement with the results obtained by Lievonen et al. [34]. However, the ζ-potential of −44 ± 4 mV in pure water is slightly less negative than the previously found value of −60 mV [34].

Additionally, Xiong et al. [38] used THF as solvent for the dissolution of lignin from an acetic acid Organosolv process. The lignin concentrations in THF ranged from 10–100 g/L. The lignin solution was subsequently added into a stirred vessel filled with water. The yielded suspension was centrifuged, repeatedly washed with deionized water and lyophilized to yield dry nanoparticles. The most suitable conditions were found to be at agitation speeds above 300 rpm, injection rates of 5–40 mL/h and lignin concentrations in THF of 10–20 g/L where higher concentrations showed increasing particle sizes. Additionally, the solvent to antisolvent ratio was varied and showed minimal average sizes for the volume ratios 1:20 and 1:10 with 151 and 161 nm, respectively. Transmission electron microscopy (TEM) images confirm the particle sizes determined by DLS (dynamic light scattering) but show the presence of agglomerates. The method is also patented and claims successful application for any other industrial type of lignin [39].

Wheat straw alkali lignin was acetylated by Qian et al. [17] prior the precipitation step. The acetylated lignin (ACL) is water-insoluble but soluble in THF. Therefore, a THF solution with 1 g/L ACL was prepared and water was gradually added into the ACL/THF solution. During this process, the scattered light intensity (SLI) was monitored. The ACL’s colloidization occurred between water contents of 44 and 67 vol %, indicated by an increasing SLI. An excessive amount of water was added to the dispersion after the completed colloidization process and THF was removed by rotary evaporation, which resulted in an average hydrodynamic radius of 110 nm determined by DLS. TEM images of dried particles showed a separation of the particle’s core and shell, indicating cores consisting of the ACL fractions with more hydrophobicity and shells consisting of the ACL fractions with less hydrophobicity, without covalent bonding between shell and core.

Further work by Qian et al. [40] produced nano- and microsized spheres in three different size ranges from pine wood OS and corn cob EHL, which were in non-acetylated and acetylated form. The precipitation methods for all three size ranges used 8:1 vol/vol acetone/water as solvent. Lignin concentrations and antisolvents were used as follows: (1) Large spheres: a lignin solution with 100 mL acetone/water and 10 g/L was gradually diluted with 400 mL of 0.1 M NaCl aqueous solution while stirring at room temperature; (2) midsized spheres: a lignin solution with 100 mL acetone/water and 10 g/L was gradually diluted with 400 ml of deionized water while stirring at room temperature; (3) small spheres: a lignin solution with 100 mL acetone/water and 0.1 g/L was gradually diluted with 400 mL of deionized water while stirring at room temperature. Large and midsized particles were centrifuged, washed with water and lyophilized, while small particles were concentrated in a rotary evaporator and lyophilized. For the non-acetylated EHL lignin, the DLS particle size analysis showed 41, 200 and 2700 nm average hydrodynamic diameters for small, midsized and large spheres, respectively. The acetylated EHL lignin showed similar results with 40, 180 and 2000 nm diameter but wider distributions. No particle sizes for OS were reported.

Furthermore, Yearla and Padmasree [41] used acetone/water solutions (9:1 vol/vol) as solvent for the commercial alkali lignin and hardwood lignin isolated by an acidic dioxane method [42]. Here, the lignin was dissolved at a concentration of 10 g/L in in acetone water solutions. The lignin solution was added rapidly to deionized water stirred at 300 rpm at a ratio of 1:2 lignin solution/water. Agglomerates were separated by centrifugation at 1500 g and scanning electron microscope (SEM) and TEM were used for determination of size and morphology. The average sizes of dioxane lignin nanoparticles and alkali lignin nanoparticles were found to be 104 ± 60 nm (SEM)/80 ± 27 nm (TEM) and 104 ± 41 nm (SEM)/82 ± 33 nm (TEM), respectively and increased only marginally after 30 days' storage at room temperature. The yield of dioxane-extracted lignin was almost twice as high (63%) when compared with the alkali lignin (33%) and was determined by centrifuging the supernatant of the first centrifugation step at 40,000 rpm.

**Figure 2:**
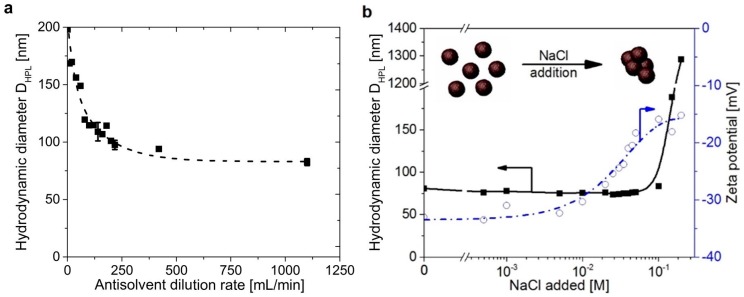
(**a**) Influence of the dilution rate on the Organosolv lignin (OS) particle diameter at initial lignin concentrations of 1 wt %; (**b**) Influence of the ionic strength on particle diameter and ζ-potential by addition of NaCl. Reprinted with permission from Richter et al. [43]. Copyright 2016 American Chemical Society.

Richter et al. [43] used acetone and water as solvent and antisolvent, respectively. OS was dissolved in acetone at concentrations of 5 g/L. The dilution/precipitation was divided into two steps: (1) Addition of 9.2 mL of water at rates from 1 to 220 mL/min into 1 mL of lignin solution; (2) rapid (1–1100 mL/min) dilution with water to a final lignin concentration of 0.05 wt %. The smallest particle sizes were achieved at dilution rates of >400 mL/min (see Figure 2a) and low initial lignin concentrations. The nanoparticle dispersions showed a stable behavior between pH 3.2 and pH 8.5 with a diameter of around 80 nm and a ζ-potential of around −45 mV. At pH values below 3.2 and over pH 12, aggregation and dissolution occurred, respectively. Additionally, the stability in dependency of the ionic strength was evaluated by adding NaCl to the dispersion and showed a stable dispersion up to a concentration of 70 mM (see Figure 2b). For certain applications, a positive surface charge of the particles is necessary, which was achieved by adsorption of PDAC on the nanoparticles’ negative surface. By using lignin to PDAC ratios of greater than 0.06 wt %, a ζ-potential above +20 mV and stable dispersions could be achieved.

**Figure 3:**
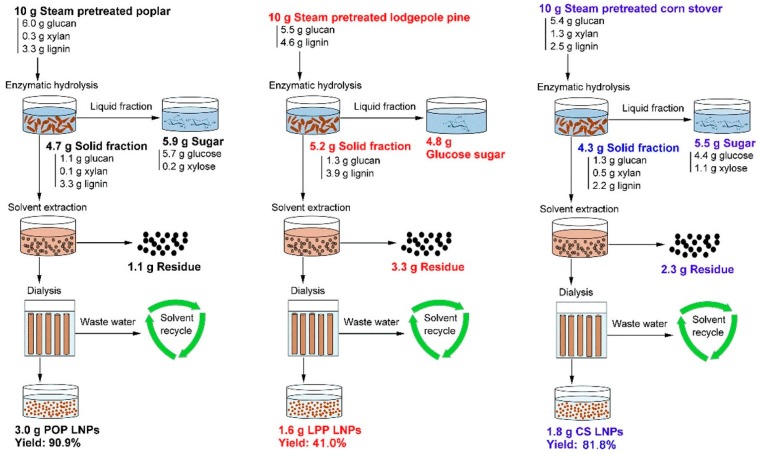
Flowchart of an integrated lignin nanoparticles production into a lignocellulosic biorefinery. Enzymatic hydrolysis was conducted for 72 h at a solid loading of 2 wt/vol % using Cellic Ctec3 (40 mg_enzyme_/g_glucan_). Lignin extraction was conducted at a very mild condition (2 wt/vol %, 3 h, 80 °C) by dimethyl sulfoxide (DMSO) before subsequent dialysis at room temperature. The dialysis was continued until no DMSO trace was detected in the wastewater. Reprinted with permission from Tian et al. [44]. Copyright 2017 American Chemical Society.

The valorization of lignin is a key factor in achieving economically viable second generation biorefineries. Therefore, Tian et al. [44] showed a lignocellusic biorefinery process chain (see Figure 3) with integrated production of lignin nanoparticles from poplar, lodgepole pine and corn stover. The raw materials were steam-pretreated [45] before enzymatic hydrolysis for 72 h. The solid residues from the enzymatic hydrolysis were collected and extracted, without drying, with DMSO under very mild conditions at 80 °C for 3 h. The solid loading during extraction was kept at 4 g/L based on the dry substrate. The resulting lignin solution was separated from the solid residues by filtration and directly transferred to a dialysis bag (12–14 kDa). The dialysis was conducted in periodically-replaced tap water and stopped when no trace of DMSO was found in the wastewater. This process resulted in average particle sizes of around 100 nm for woody biomass and 200 nm for agriculture residues, which showed stable behavior in water at pH values between 4 and 10. However, the SEM images showed a strong tendency of agglomeration for nanoparticles from corn stover. This phenomenon indicated that corn stover lignin NPs might contain more hydrophilic groups, which can be associated with carbohydrate impurities [17]. The determination of residual carbohydrates in the nanoparticles proved this assumption and showed total carbohydrate contents of 0.68, 1.63 and 10.57 wt % for poplar, pine and corn stover-originating nanoparticles, respectively. It is important to mention the differences in nanoparticle yields, which are 90.9% for poplar, followed by 81.8% for corn stover and only 41.0% for pine based on the lignin content after enzymatic hydrolysis.

#### 2.1.2. Hollow Particles

Open-mouthed hollow nanocapsules have recently gained great interest due to their enhanced uptake capacity, diffusivity and catalytic performance [46,47,48,49]. Moreover, particles with controllable cavities and openings have potential value in selective encapsulation [50].

**Figure 4:**
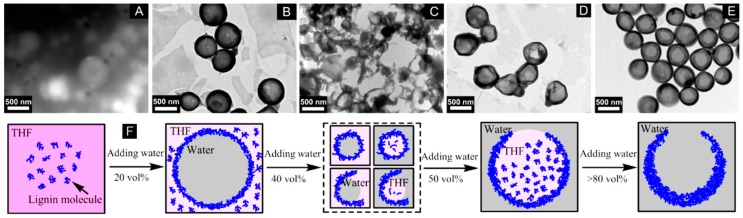
Transmission electron microscopy (TEM) images of the samples obtained from the dispersions at different water contents at an initial lignin concentration of 0.5 g/L. Water content: (**A**) 0 vol %, (**B**) 20 vol %, (**C**) 40 vol %, (**D**) 50 vol %, (**E**) >80 vol %, after removing tetrahydrofuran (THF). (**F**) Schematic representation of the lignin hollow nanosphere’s formation process in THF/H_2_O. Reprinted with permission from Xiong et al. [51]. Copyright 2017 American Chemical Society.

Xiong et al. [51] were able to produce these open-mouthed hollow nanocapsules (see Figure 4E) by precipitation of EHL/THF solutions with the addition of water. EHL/THF solutions were prepared at concentrations of 0.5, 1, 1.5, and 2 g/L and used in 10 mL portions. Under stirring at 600 rpm and room temperature, 40 mL of deionized water were dropped into the EHL/THF solution at a rate of 2 mL/min. The particle size determined by DLS showed increasing particle sizes, from 419 nm to 566 nm, with an increasing initial EHL concentration from 0.5 g/L to 2 g/L. The hollowness of the particles was investigated by TEM and revealed the opening’s decreasing diameter (1.5–14 nm for 2 g/L; 15–60 nm for 0.5 g/L) and increasing shell wall thickness with an increase in the initial EHL concentration. This precipitation process does not differ greatly from the method described previously by Xiong et al. [38] using THF/water as solvent/antisolvent but using lower initial lignin concentrations and higher addition rates, which lead to hollow particles. Figure 4 shows the precipitation process at different water contents monitored by TEM. The behavior is explained by a phase separation between analytical-grade purity THF and water. Usually, THF/water is miscible but it is suggested that small impurities in the analytical-grade purity THF form miniemulsions [51]. This assumption was confirmed by using HPLC-grade THF for a comparison where HPLC-grade THF yielded solid spheres and analytical-grade purity THF hollow spheres.

Li et al. [52] prepared open-mouthed hollow spheres by using KL and THF/water as solvent/antisolvent, where the THF was analytical grade. 3.0 mL of KL/THF stock solution was first added to a serum bottle; next, ultrapure water was added to the bottle dropwise at a rate of 1.2 mL/min while continuously stirring until the water content reached 90 wt %. The DLS results reveal two different particle size fractions with diameters of 100–200 nm for the smaller particle fraction and 500–600 nm for the larger particle fraction. The initial lignin concentration’s variation yielded smaller particle sizes and thinner shells for low concentrations and larger particle sizes and thicker shells with increasing initial lignin concentration. These results are consistent with the findings of Xiong et al. [51].

Li et al. [53] used KL in a solvent/antisolvent system of dioxane/water. Ultrapure water was added to the lignin/dioxane solution at rates ranging from 0.7 to 14 mL/min with decreasing particle sizes at higher rates. An initial lignin concentration in dioxane of 3.69 g/L with a water dropping rate of 3.3 mL/min yielded spheres with a hollow characteristic and an average hydrodynamic radius of 145.5 nm.

In another study, Li et al. [54] used ethanol as solvent for KL and water as antisolvent. The KL concentration in ethanol was set to 2.285 g/L and ultrapure water was added under stirring at dropping speeds ranging from 0.456 to 36 mL/min until the water content reached 90 wt %. Increasing dropping speeds resulted in smaller capsule diameter ranging from around 60 to 350 nm. However, this preparation method produced closed capsules that do not open.

### 2.2. pH Shifting

**Figure 5:**
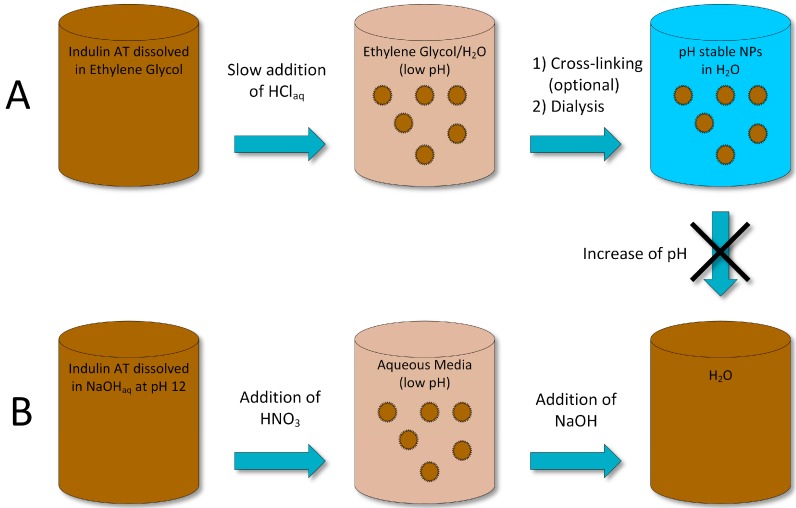
Schematic illustration of the two different precipitation methods. The canceled arrow between method (**A**) and (**B**) indicates the stability of particles at increased pH value produced with method (**A**). Precipitation of ethylene glycol lignin solution with HCl and following optional crosslinking and dialysis. (**B**) Precipitation of lignin from aqueous NaOH solution at pH 12 by dropping the pH value with HNO_3_. Adapted from Frangville et al. [16]

Frangville et al. [16] introduced the “standard methods” of lignin nanoparticle precipitation, which were used for several other publications [23,43,55,56,57,58,59,60,61,62,63]. The first standard method is based on simultaneous pH and solvent change and the second on a pH change from basic to acidic in aqueous medium [64]. These two methods result in nanoparticles with significantly different properties and stability. The method is based on the fact that low-sulfonated lignin is practically insoluble in water under neutral and acidic conditions [65]. Conversely, a significant solubility is given in ethylene glycol and water above pH 10. Using this property, two methods were developed for lignin NP production where the first uses ethylene glycol as solvent and precipitation occurs by adding aqueous HCl to the solution followed by an optional crosslinking step and dialysis with water. The second method uses water set to pH 12 with NaOH as solvent for lignin and precipitation is performed by dropping the pH value using HNO_3_ [16]. Both methods are illustrated in Figure 5.

To elaborate, the first method introduced by Frangville et al. [16] used solutions of Indulin AT lignin in ethylene glycol. Subsequently, certain amounts of aqueous HCl solution were added to 50 mL lignin solution. The resulting suspension was dialyzed in water for three days. When crosslinking was applied, 150 mL 0.2 wt % aqueous glutaraldehyde was added to a 50 mL nanoparticle suspension prior dialysis. During the experiments, the ratio of HCl to lignin solution, HCl concentration, initial lignin concentration and the HCl’s dosing rate of HCl were varied. DLS results show all four varied parameters had dependencies on the particle size. Higher ratios of HCl to lignin solution and higher HCl concentrations show increased particle sizes whereas slower dosing rates decrease the average diameter of the particles. Varying the initial lignin concentration shows maximum particle sizes at an initial concentration of 1.5 wt %. Therefore, optimal preparation conditions were proposed with an HCl concentration of 0.025 M, initial lignin concentration of 0.56 wt %, a dosing rate of 0.04 mL/min and a final HCl solution concentration of 15 vol % resulting in an average particle size of around 100 nm. Stability tests showed negligible changes after 30 days storage in water in the pH range of 6–9, even for particles without crosslinking. The ζ-potential of lignin particles without crosslinking treatment in the pH range of 6–9 showed values between −40 and −30 mV.

The second method using the base/acid-precipitation method resulted in a particle mean diameter of around 71 nm. However, the particles show a poor storage stability as they were only stable at pH 2.1 even with the addition of glutaraldehyde as a crosslinker.

**Figure 6:**
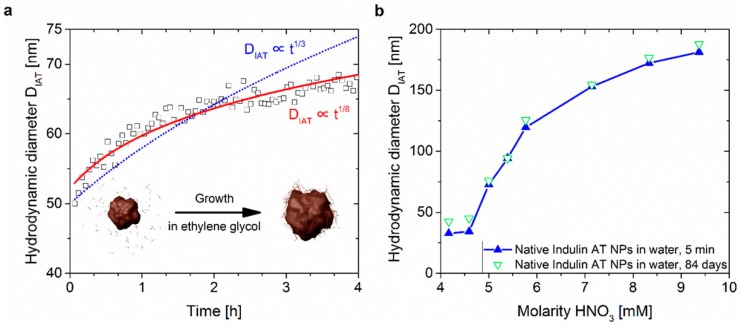
(**a**) Growth kinetics after the initial pH drop with a final HNO_3_ concentration of 5 mM; (**b**) Hydrodynamic diameter versus final concentration of HNO_3_ after the pH drop. Reprinted with permission from Richter et al. [43]. Copyright 2016 American Chemical Society.

Richter et al. in [43,56] used the identical ethylene glycol precipitation method and raw material as mentioned above [16]. The investigated process parameters and storage stability were extended by the precipitation temperature variation from 25 °C to 50 °C. The results show a clear tendency of increasing particle sizes with increasing precipitation temperature, which was proposed to be contributed by the increased solubility and larger rate of polymer self-diffusion at higher temperatures. Additionally, the particles’ growth was monitored after the pH drop using DLS to describe the rapidly formed nuclei’s growth kinetics during the pH drop. Growth kinetics of nanoparticles show a commonly exponential increase [66,67] as

(1) $$D\propto t^{\frac{1}{x}}$$

where D represents the hydrodynamic particle diameter at time t. The exponential coefficient x is dependent on the growth mechanism’s limiting step. The x found for the growth rate at 25 °C is ~8. Hence, it is much slower than diffusion limited (x=3) or surface reaction limited (x=2) growth mechanisms after the Lifshitz−Slyozov−Wagner (LSW) model [68]. Figure 6a shows the plotted estimated growth curve in ethylene glycol, which is in good accordance with the experimental data. By comparison, the estimated growth curve of a diffusion limited growth kinetic is also shown in the plot. Figure 6b shows the hydrodynamic diameter’s dependency on the final HNO_3_ concentration measured in water after 30 s and 84 days after the pH drop. The hydrodynamic diameter clearly increases with increasing final HNO_3_ concentration and all samples show a good storage stability [43].

Gupta et al. [55] used an adapted version of the method mentioned above using commercially-available alkali lignin (PB 1000) dissolved in ethylene glycol and precipitated using aqueous HCl. The yielded dispersion was subsequently dialyzed with water for 72 h. The precipitation process investigated varying the initial lignin concentration, concentration of HCl, dosing rate of HCl and concentration of HCl after the pH drop. The variation of these parameters showed a similar influence on the final particle size as shown by Frangville et al. [16]. However, a minimum particle size of around 125 nm was found for a dosing rate of one drop every 30 s and particle sizes increased with increasing and decreasing dosing rate. Additionally, the particles produced by this method showed excellent storage stability with minor changes in particle size after three weeks. Chemical analysis of the NPs showed that the ash content was reduced in comparison to the raw material from 13–14% to 1.4–1.8%. However, information in this literature is not fully reproducible and has, therefore, to be handled with caution.

Yang et al. [23] used a partially-adapted method in several publications [57,60,61,63]. The raw material used was lignin residue of Arundo donax L. after steam explosion treatment and enzymatic hydrolysis. The lignin/ethylene glycol suspension was prepared at a concentration of 4 wt %. In the precipitation step, 10 mL of 0.25 M HCl was added to a 192 mL lignin solution at 35 °C and a rate of 2 drops/min. The reaction was retained for 2 h. Where Frangville et al. [16] filtered the ethylene glycol solution to remove insoluble components before precipitation, Yang et al. [23] perform the filtration after precipitation (22 µm pore diameter) and claim to obtain higher yields (approximately 10%) and more homogeneous NPs. After dialysis, the SEM image analysis revealed an average diameter of 48.85 ± 16.38 nm and no particles below 20 nm or above 80 nm were observed.

Higher particle diameters were obtained by Ge et al. [59] using alkali lignin and both methods introduced by Frangville et al. [16], but substituting the dialysis step by centrifugation. DLS analysis showed average diameters of 375 ± 18 nm and 278 ± 13 nm for the ethylene glycol and acid/base method, respectively. However, stability of the particles was not investigated.

Li et al. [58] assert that nanoparticles with a size distribution of 50–280 nm can be produced from purified lignin dissolved in an alkaline solution, where the bases NaOH, KOH and ammonia can be used. The precipitation with sulfuric acid, nitric acid or hydrochloric acid at a certain speed and a subsequent freeze-drying result in NPs with good dispensability and adsorption properties. No stability information was published that could verify the results by Frangville et al. [16] about low stability particles of this method.

Wei et al. [69] used alkali lignin from furfural residues by dissolving them in water set to a pH of around 11 with concentrated ammonia solution and precipitation was carried out by dropping the pH to 3 by adding HCl. This method resulted in an average particle diameter of 182 nm and the pH-dependent stability was used for the fabrication of pH-responsive Pickering emulsions.

The “standard methods” introduced by Frangville et al. [16] were repeated by several other researchers, which verified their applicability for different types of lignin, especially for the ethylene glycol method. As mentioned for the solvent shifting methods, particle size can be controlled by the precipitation conditions. However, also this method poses issues in terms of industrial applicability due to high solvent demand and low yields around maximal 10% [59].

### 2.3. Crosslinking/Polymerization

Several methods were used to obtain solid and hollow lignin NPs. Because methods can be quite different between those yielding solid and hollow particles, these methods are reported separately in the following chapters.

#### 2.3.1. Solid Structures

KL particles were synthesized in water-in-oil microemulsions by Nypelö et al. [70]. The purified KL was dissolved in a 1 M aqueous NaOH solution. The oil phase was comprised of octane and a surfactant mixture containing sorbitan monooleate, polyoxyethylen(20)-sorbitan-monooleate and 1-pentanol at a volume ratio of 2:1.2:0.9. The microemulsion was prepared by adding the lignin solution to octane containing the surfactant mixture and gently turning upside-down. Crosslinking was achieved by adding epichlorohydrin under gentle stirring at 55 °C for 40 min. The oil was removed by the repeated addition of excess water and centrifugation. The emulsion formulation and composition of the internal phase, surfactant and crosslinker concentrations were used to control the size in a range from 90 nm to 1 µm and the integrity of the resulting particles.

Popa et al. [71] 2011 utilized wheat straw and Sarkanda grass lignins isolated by alkaline treatment as well as three commercially-available alkali lignins (PB 1000, PB 2000 and PB 3000; Granit Recherche, Switzerland). The synthesis of nanoparticles was conducted by hydroxymethylation [72,73] and epoxidation [74]. Briefly, hydroxymethylation was conducted by dissolving lignin in an aqueous NaOH solution and reaction with formaldehyde using NH_4_OH as catalyst based on patent [75]. The resulting product was recovered by a pH drop precipitation and centrifugation. The epoxidation took place in an aqueous NaOH lignin solution by adding epichlorohydrin and subsequent separation by centrifugation. The epoxidation of the commercial lignins resulted in nanoparticles with average diameters ranging from 246 to 248 nm and hydroxymethylated lignin particles are in the dimensional range of 50 to 800 nm.

Gîlcă and Popa [21] repeated the epoxidation as mentioned above at optimum conditions and yielded nanoparticles with average particle diameters between 70 and 200 nm, where PB 3000 showed the smallest particle sizes and PB 2000 the biggest sizes. The particle size of PB 1000 fell between the other NPs and therefore shows no evidence of a molecular weight dependency.

Reversible addition-fragmentation chain transfer (RAFT) allows for a high precision synthesis of polymer-grafted NPs which have numerous applications ranging from lubrication to improved composite materials [76,77]. Therefore, this method was used for polymer-grafted lignin NPs synthesis by Gupta and Washburn [78] and Silmore et al. [79] from KL. The identical procedure was used in both publications where [78] is more focused on the NPs and [79] on the behavior of the NPs in emulsions. The synthesis was comprised of two steps: (1) Preparation of RAFT Macroinitiator; (2) grafting. The lignin Macroinitiator made by reaction of lignin with xanthanate carboxylic acid where the ratio between both reactants affects the graft density. The grafting itself took place by mixing the RAFT Macroinitiator with azobis-(isobutyronitrile) and a monomer (acrylamide or acrylic acid) in DMF and reaction at 70 °C for 24 h. The variation of graft density, degree of polymerization and type of used monomer resulted in nanoparticles with average diameters ranging from ~15 nm to ~367 nm.

#### 2.3.2. Hollow Structures

The synthesis of micro- and nanocapsules was conducted in an oil/water-interface using micro- or miniemulsion polymerization/crosslinking which allow for the encapsulation of hydrophilic and hydrophobic components in amphiphilic lignin [80,81].

**Figure 7:**
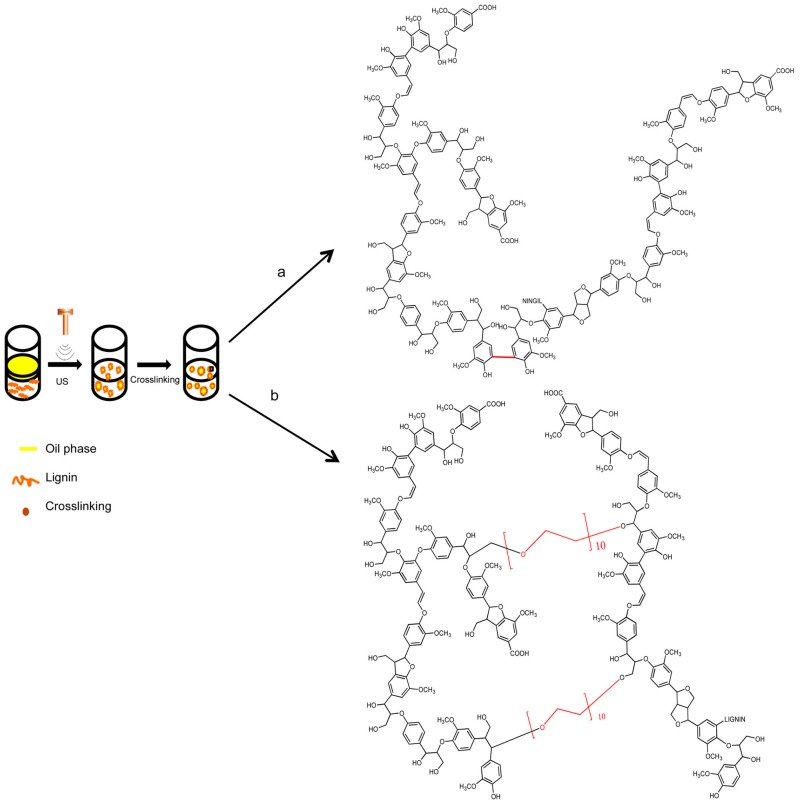
Schematic representation of the production method and of crosslinking routes. Route (**a**) No additional crosslinking agent; route (**b**) cross-inking by addition of PEGDEG. Reprinted with permission from Tortora et al. [20]. Copyright 2014 American Chemical Society.

Oil-filled KL microcapsules were prepared by Tortora et al. [20] by first creating an oil-in-water emulsion followed by a high-intensity, ultrasound-assisted crosslinking of lignin at the water/oil interface (see Figure 7). In this work, three different microcapsule preparation methods were used: (1) Capsule preparation without additional crosslinking agent; (2) capsule preparation in the presence of H_2_O_2_; (3) capsule preparation in the presence of poly(ethylene glycol) diglycidyl ether (PEGDEG). All three methods essentially use an aqueous lignin solution forming an emulsion with olive oil and crosslinking during sonication with a high-intensity ultrasonic horn. In methods 2 and 3, H_2_O_2_ and PEGDEG, respectively, were added to the olive oil before emulsification. The mechanism responsible for the formation of lignin micro capsules combines two ultrasound-induced phenomena: emulsification and crosslinking. The amphiphilic lignin chains are thought to diffuse toward the oil microdroplets in the emulsion and to stabilize the water/oil interface. The crosslinking without the addition of agents (method 1, see Figure 7a) uses hydroxyl and superoxide radicals generated during the acoustic cavitation process to induce crosslinking between the lignin chains’ phenolic groups [82,83]. Method 2 uses the addition of H_2_O_2_ to increase the concentration of hydroxyl radicals and is therefore meant to support the crosslinking. In method 3 (see Figure 7b), PEGDEG is added before emulsification, where the surfactant and crosslinking properties may play a role in improving the lignin’s encapsulation ability [84,85]. The different methods showed a successful preparation of micro/nanocapsules with a spherical shape and average diameters in the range of 0.3 to 1.1 µm.

A similar approach was used by Yiamsawas et al. [35]. Briefly, a water-soluble sodium lignosulphonate fraction was used to form lignin–polyurea/polyurethane nanocontainers at the interface of stable water nanodroplets in a reverse miniemulsion. An enzyme-responsive, cleavable crosslinked shell surrounding a liquid aqueous core was generated by selective polyaddition using toluene diisocyanate as crosslinking agent. The gained nanocontainers showed a high stability over a period of several weeks when redispersed in water. The average capsule wall thickness for all dry samples was found to be between 10–20 nm with diameters of 311 to 390 nm when dispersed in water.

**Figure 8:**
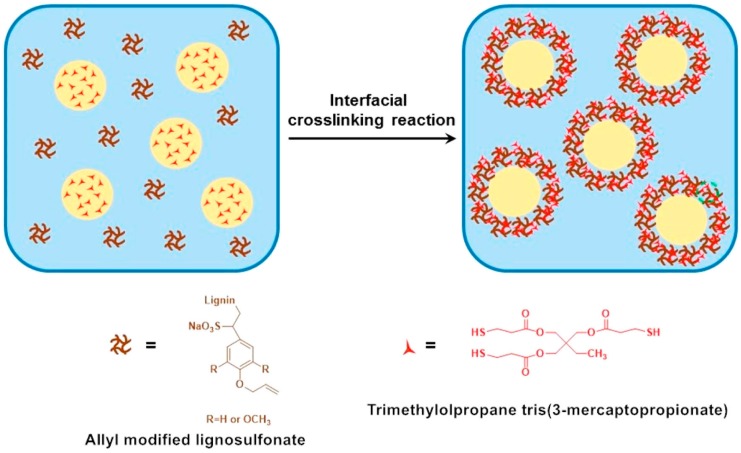
Preparation of lignin-based Nanocapsules via interfacial miniemulsion crosslinking reaction. Reprinted with permission from Chen et al. [86]. Copyright 2014 American Chemical Society.

Chen et al. [86] synthesized pH-responsive lignin-based nanocapsules for the controlled release of hydrophobic molecules. Lignosulfonate was first grafted with allyl groups through etherification. The modified lignosulfonate was further dispersed in an oil-in-water miniemulsion using sonication. At the interfaces of the miniemulsion droplets, the shell was formed via a thiol-ene radical reaction of the allyl-functionalized lignin and the thiol-based crosslinking agent (see Figure 8). The nanocapsules’s particle sizes could be fine-tuned by controlling the process parameters and they ranged from approximately 100 to 400 nm. The capsules could effectively embed hydrophobic cargo via miniemulsion.

**Figure 9:**
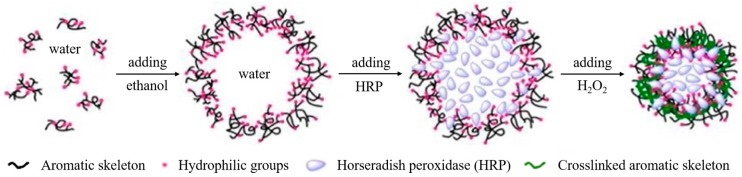
Lignosulfonate vesicular reverse micelles self-assembly in ethanol/water with encapsulated horseradish peroxidase and crosslinking with H_2_O_2_. Reprinted with permission from Zhong et al. [87]. Copyright 2014 American Chemical Society.

Zhong et al. [87] used sodium lignosulfonate purified by ultrafiltration in two methods for the production of nanocontainers. The first method simply comprised aqueous lignosulfonate solutions to which ethanol was added gradually by stirring under room temperature. At an ethanol concentration of around 63 vol %, emulsification and the formation of nanocontainers started. The obtained particles showed a hydrodynamic diameter of 200 nm but a solid structure. However, after six days of storage, the diameter increased to 240 nm and due to strong electrostatic repulsion, particles became hollow. The shell thickness could be easily adjusted by adding different concentrations of NaCl to the initial lignin solution. The second method (see Figure 9) is identical to the first method but horseradish peroxidase was added to the nanocontainer suspension resulting from the first method that was encapsulated. The addition of H_2_O_2_ started a crosslinking reaction in the lignin and stabilized them.

### 2.4 Other Formation Methods

There are several other techniques for preparing nanostructured materials that have not been extensively investigated regarding the application on lignin. Therefore, this chapter provides an overview of the techniques showing partially promising characteristics.

#### 2.4.1. Mechanical Treatment

Mechanical treatments like dry and wet milling techniques are widely used to reduce particle size down to nanometer scale [88,89,90]. The milling process has the disadvantage of non-uniformity in particle size and broad particle size distributions [91] but is still a simple process for nanoparticle production.

Nair et al. [92] used a simple mechanical homogenization method to create lignin nanoparticles. Softwood KL water dispersions at a concentration of 5 g/L were treated for up to 4 h with a high shear homogenizer at 15,000 rpm. The particle sizes were determined by SEM and showed for the starting KL that 50% of the particles were larger than 500 nm, with some particles as long as 6–7 µm in the initial KL. After 2 h of shearing, 75% particles had diameters less than 100 nm. The size distribution became narrow with increasing mechanical shearing duration. After 4 h of mechanical shearing, 100% of the lignin particles were homogenized to sizes less than 100 nm and a narrow particle size distribution. The ^31^P NMR data revealed that no changes in the chemical structure and no change in the molecular weight happened during the 4 h of mechanical treatment.

Gilca et al. [36] used sonication for the size reduction of wheat straw and Sarkanda grass lignin. In contrast to the sonication carried out by Tortora et al. [20], which caused crosslinking and an increase in molecular weight, this sonication process resulted in decreasing molecular weight and is roughly considered as mechanical treatment. Briefly, aqueous suspensions of 0.7% lignin concentration were treated with an ultrasonic horn at 20,000 kHz frequency and 600 W power. The average particle diameter could be significantly reduced from 1–10 µm to 10–20 nm for both types of lignin. Additionally, Zimniewska et al. [93] used ultrasonication and could achieve lignin particles with diameter less than 20 nm determined by TEM.

Methods patented by Shawn et al. [94] and Zhiming et al. [95] assert a size reduction down to 10 nm by ball milling and milling at −18 °C, respectively.

#### 2.4.2. Ice-Segregation-Induced Self-Assembly

Ice-segregation-induced self-assembly (ISISA) describes a technique that involves the dissolution or suspending of polymeric and monomeric precursors in water and freezing of the solution. As the solution freezes, the polymeric material gets displaced and phase separated by the growing ice crystals in an orderly manner, essentially templating the polymer around the ice crystals [96].

**Figure 10:**
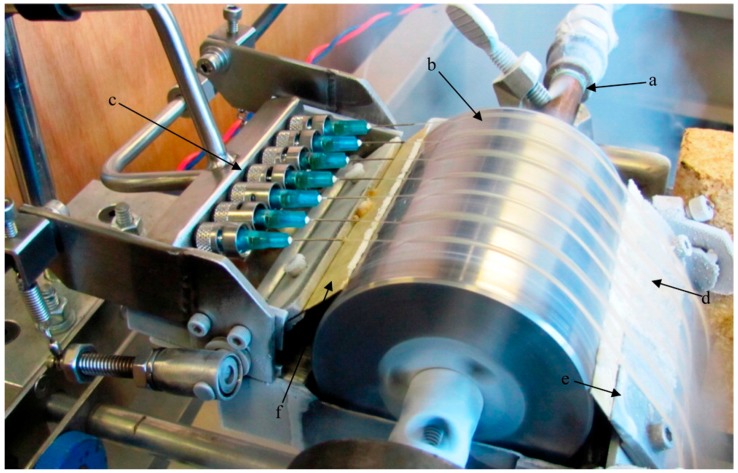
Image of the rapid freezing device for polymeric nanofiber production. (**a**) Liquid nitrogen supply (**b**) Steel drum rotating at ∼300 rpm; (**c**) Needles delivering lignin solution to the drum; (**d**) Ribbons of frozen solution containing the templated nanoscale lignin matrix; (**e**) Blade to ensure release of the frozen ribbons; (**f**) Spring tension-loaded wiper blade regulating the thickness of the liquid nitrogen layer present on the drum surface. Reprinted with permission from Spender et al. [97]. Copyright 2012 American Chemical Society.

Spender et al. [97] used this technique to produce nanofibers from alkali lignin as a precursor of carbon nanofibers. Figure 10 shows the experimental setup, which was developed for this purpose. It is essentially comprised of a drum rotating at around 300 rpm, which is cooled to 77 K with liquid nitrogen and a needle manifold delivering the lignin solution as a thin film, not greater than 130 µm on the drum.

**Figure 11:**
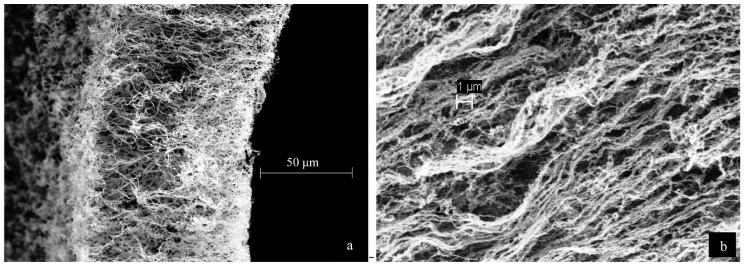
Scanning electron microscopy (SEM) images of lyophilized lignin ribbons, (**a**) Indulin AT nanofibers based on a 0.1% *w*/*w* solution, (**b**) Reax 85A lignin nanofibers based on a 0.3% *w*/*w* solution. Reprinted with permission from Spender et al. [97]. Copyright 2012 American Chemical Society.

The thin film allowed for the solution’s rapid freezing, which occurred within approximately 0.01 s of initial contact with the drum. The frozen ribbons of lignin solution were lyophilized afterwards. SEM imaging revealed fibrous structures and single fibers with diameters of 100 nm or less and lengths typically greater than 10 μm as shown in Figure 11.

Mishra and Wimmer [98] used a similar procedure to obtain colloidal lignin particles. Dioxane soluble alkali lignin was dissolved in DMSO at four different concentrations (0.02, 0.07, 0.1 and 0.5 wt/vol %) and sprayed on a liquid nitrogen pre-cooled copper plate by using an ultrasonic nebulizer and nitrogen as carrier gas. The frozen droplets were transferred in water at pH 10.5 and kept there overnight. The yielded suspension was dialyzed against water at pH 10.5 for 72 h. SEM and TEM imaging revealed that initial lignin concentrations of 0.02 wt/vol % lead to open-mouthed hollow nanocapsules, while higher concentrations lead to solid particles. The particle sizes are consistent with this finding because particle sizes generally increase with increasing lignin concentration except in the transition from hollow to solid particles. Therefore, the SEM images show an average diameter of around 130 nm for the hollow particles at initial concentrations of 0.02 wt/vol % but only around 80 nm for the solid particles at initial concentrations of 0.07 wt/vol %.

#### 2.4.3. Template-Based Synthesis

Many methods for preparing nanomaterials have been developed for simple particle shapes, but not for specially-tailored structures. If a desired shape and morphology of the final product is needed, the “template synthesis” method provides a route for enhancing the nanostructural order. This method uses a template with a desired shape to synthesize the final micro- or nanostructured product on its surface. The nanostructures can remain on the template surface or can be freed and collected as free nanostructures [99].

Caicedo et al. [100] and Ten et al*.* [101] used sacrificial alumina membranes with a nominal pore diameter of 200 nm as a template for the synthesis of lignin-based nanotubes and nanowires. Caicedo et al. [100] used lignin extracted form maize brown midrib1 (bm1) stover containing lignin with increased levels of coniferaldehyde due to its mutation [102]. The template membranes used were treated to increase the number of reactive hydroxyl groups on the surface and subsequently activated with (3-aminopropyl)-triethoxysilane (APTES). The prepared membranes were immersed in a lignin solution containing sodium phosphate buffer pH 7.4 with 1M NaCl for 24 h at 4 °C where lignin was attached via a Schiff´s base reaction. A dehydrogenation polymer (DHP; “synthetic lignin”) was deposited on the resulting coating via oxidative coupling catalyzed by horseradish-peroxidase using hydroxycinnamyl alcohols (monolignols), hydroxycinnamylaldehydes or hydroxycinnamic acids, either neat or in mixtures. To release the nanostructured material, the alumina template was immersed in 5 vol % phosphoric acid containing 0.5% surfactant and dissolved completely. This procedure resulted in bundles of nanowires and nanotubes due to material deposition on the top and bottom of the membrane surfaces. Hence, the surfaces were sputter-coated with Au/Pd prior to functionalization, which makes them inert to the reaction with APTES. The adapted method leads to individual nanotubes, which could be investigated using TEM and SEM. The structures’ outer dimensions are known due to the membrane templates’ given dimensions. Therefore, the evaluation was focused on the wall thickness. The images revealed that wall thickness was greater with the higher proportion of p-coumaric acid in the reaction, while a higher proportion of ferulic acid resulted in hollow nanotubes. Hence, the structural characteristics can be simply controlled by the chemical composition.

Ten et al. [101] used an identical procedure and template membrane dimensions as described previously [100] but used lignins from different sources and isolation methods. The lignins used were isolated from sugar cane bagasse, poplar, loblolly pine, sorghum and a mutation of sorghum (BTx623-bmr6), which has reduced S-residues and an increased cinnamaldehyde end-groups content in the lignin [103]. The isolation methods include the Klason lignin method [104], Thioglycolic acid method [105], isolation with NaOH solution [106] and isolation of lignin from sugar cane bagasse using phosphoric acid [101]. Length, diameter and wall thickness were measured by analyzing SEM images and the aspect ratio was calculated based on the length and diameter results. All results are listed in Table 1, revealing that the nanotubes’ lengths vary primarily as a function of lignin isolation procedure rather than the species from which the lignin originated. The calculated aspect ratios show a substantially higher value for the Thioglycolic acid isolation method in comparison to the NaOH method.

Jiang et al. [107] used the lamina of montmorillonite (MMT) as a plane template to anchor cationic lignin (CL) on its two sides yielding in the formation of cationic lignin-MMT hybrid materials. Industrial lignosulphonate was purified [108] prior to synthesis with cationic lignin [109,110]. The synthesis took place in a stirred beaker at 50 °C for 48 h by simply mixing Na^+^-MMT aqueous suspensions with a concentration of 2% with CL solutions at different concentrations. The initial CL/MMT mass ratios was varied from 0.1:1 to 8:1 at pH values of 3.7 and 12. The obtained hybrid materials were subsequently separated by filtration and centrifugation. The results of the X-ray diffraction (XRD) experiments revealed the thickness of the CL layer on MMT platelets where the MMT platelets themselves have a thickness of 0.96 nm. For CL/MMT mass ratios of 0.1:1 and 0.2:1, prepared at pH 7 and 3, the CL layer showed a thickness of 0.57–0.61 nm. If the ratio was increased to 1:1, the layer thickness increased to 1.93–2.70 nm. TEM and AFM measurements could sufficiently confirm that MMT can be completely exfoliated into individually dispersed nanosheets, as plane templates to anchor two layers of disk-like CL, when the CLM hybrid materials with mass ratio ≥2:1 are prepared at pH of 3 and 7.

#### 2.4.4. Aerosol Processing

Using aerosol technology, particles can be directly produced within a desirable particle size range with consistent and controlled properties in a simple single-step continuous process [111]. Further advantages of this technology are the absence of liquid byproducts, the simplicity, the less expensive particle collection and high product yields [112,113].

**Figure 12:**
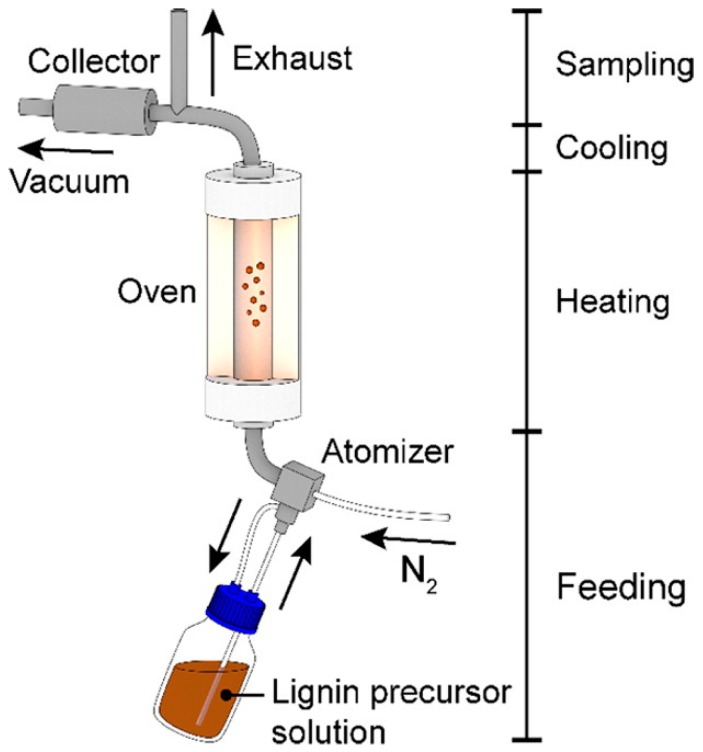
Schematic aerosol following reactor setup used for lignin particle synthesis. Reprinted with permission from Ago et al. [114]. Copyright 2016 American Chemical Society.

Ago et al. [114] used an aerosol reactor (see Figure 12) for the synthesis of lignin particles in the size range of 30 nm to 2 µm including a fractionation step. The process is comprised of a continuous atomizer, which forms an aerosol out of the lignin precursor solution, a heating tube passed by the aerosol with the aid of a gas carrier and a Berner-type low-pressure impactor for the collection and fractionation of the dry particles [115]. KL, alkali lignin and beech wood OS were used for synthesis. The precursor lignin solutions had concentrations of 0.5%, 1% and 2%, where pure water was used as solvent for alkali lignin and dimethylformamide (DMF) for KL and OS. The atomizer used was a collision-type jet atomizer operated with nitrogen [111] whose droplets were suspended at a nitrogen gas flow rate of 3 L/min. The aerosol was carried through the heated reactor with a laminar flow at 100 °C when water was used as solvent or at 153 °C when DMF was used as solvent. The solid particles were subsequently cooled and diluted with an air flow volume of 30 L/min to achieve turbulent flow prior to collection. The collector was comprised of 10 stages with nominal cutoff diameters (D50) ranging from 31 nm to 7.817 µm. The yields achieved were generally >60% based on consumed precursor solution after running the reactor for the given time.

**Figure 13:**
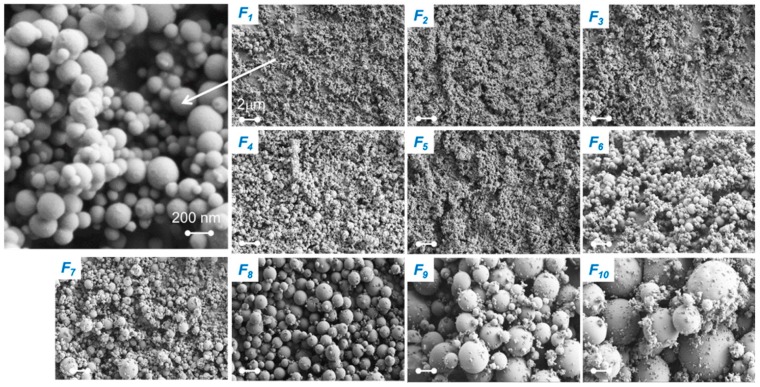
SEM images of different Kraft lignin (KL) particle fractions. F1-10 indicates fraction and the scale bar is 2 µm Reprinted with permission from Ago et al. [114]. Copyright 2016 American Chemical Society.

The fractional yield of larger alkali and OS particles (5 stages with the highest cutoff diameter) increased with increasing concentration of lignin in the precursor solutions, which can be explained by the changing apparent viscosity in the solution, increasing the droplet size produced by the atomizer [116]. Generally, the particle size distribution became broader when the mean particle size shifted to lower values. SEM and TEM images showed solid spherical particles with smooth surfaces (see Figure 13) for all three kinds of lignin and the particles’ morphology particles was preserved after redispersion in different media under heating or shear [114].

#### 2.4.5. Electrospinning

**Figure 14:**
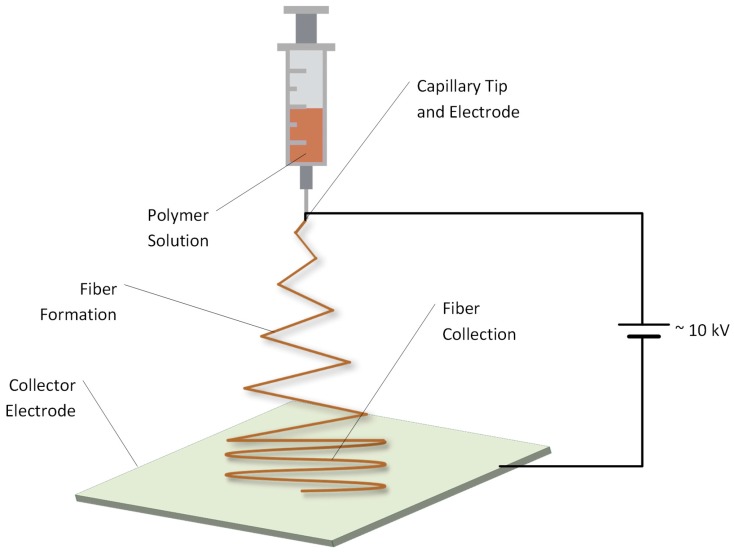
Schematic representation of an electrospinning setup.

Electrospinning is a common and well known process able to produce fibers from polymer solutions a diametric size range of nanometers to micrometers [117,118,119]. A typical laboratory scale experiment setup is shown in Figure 14 where a polymer solution is pumped through a nozzle with an inner diameter in the size range of 100 µm. The nozzle serves simultaneously as an electrode and between counter electrode and nozzle; typically, an electric field between 100 and 500 kV/m is applied. The distance between the nozzle and counter electrode, where fibers are collected, is typically between 10 and 25 cm in laboratory setups [119].

Considering that technical lignins have heterogeneous, branched structures and are degraded to low molecular weight particles during biomass delignification, these lignins tend to electrospray rather than forming continuous fibers. However, by dampening instabilities, technical lignins also have the ability to form fibers by electrospinning [120].

Dallmeyer et al. [120] used seven different technical lignins to evaluate their suitability for electrospinning. The technical lignins test set included soft- and hardwood KL, soft- and hardwood OS, sulfonated KL, lignosulfonate and pyrolytic lignin derived from bio-oil. Lignin solutions were prepared at concentrations between 10 and 50 wt % by using pure water as solvent for the lignosulfonate and sulfonated KL and DMF for other types of lignin. Despite many prepared combinations of lignin types and concentrations, none were capable of fiber formation but produced electrospray. However, adding Poly(ethylene oxide) (PEO), which facilitates biopolymer electrospinning [121,122,123], improved the spinnability. The addition of 1 wt % PEO to the spinning solution leads to uniform fibers with diameters ranging from ~700 nm up to ~1.6 µm for different types of lignin, where the fiber diameter can be adjusted by changing lignin concentrations in the spinning solution.

Ruiz-Rosas et al. [124] used OS/platinum/ethanol solutions to spin fibers as a precursor for submicron carbon fiber production. A special cone setup was used in this work. While the solution arrives through the central capillary tip, pure ethanol flows by the outermost, feeding the Taylor cone [125] with enough solvent to compensate for losses from evaporation and avoiding the solidification of the Taylor cone. This setup resulted in fiber diameters ranging from 800 nm to 3 µm. Notably, typically not-undesired electrospray and the controlling of inner and outer flowrates could allow for the encapsulation of core liquids [126,127].

**Figure 15:**
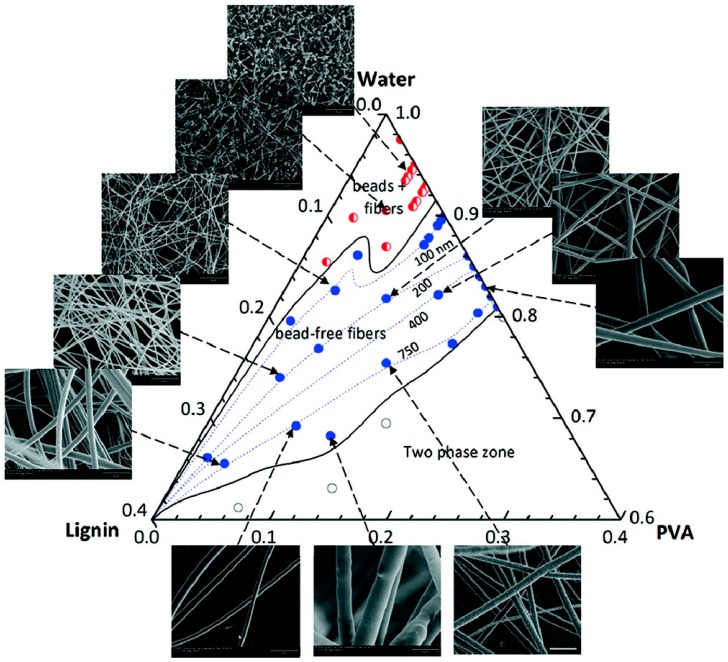
Ternery diagram showing the respective fiber morphology of the initial spinning solution composition where red circles indicate beading, blue circles indicate bead-free fibers, and unfilled white circles indicate a phase separation zone (not suitable for electrospinning). Representative SEM images are added around the ternary diagram and they include size bars of 5 μm. Reprinted with permission from Ago et al. [129]. Copyright 2012 American Chemical Society.

Lignin nanofiber reinforced with cellulose nanocrystals could be obtained by Ago et al. [128] using softwood KL. The spinning solution was comprised of water, lignin and poly(vinyl alcohol), where electrospinnable compositions can be seen in Figure 15. The spinning resulted in fiber diameters ranging from 61 ± 3 nm to 500 ± 34 nm depending on the lignin concentration. Defect-free nanofibers with up to 90 wt % lignin were achieved and up to 15 wt % cellulose nanocrystals could be incorporated, which improved the yielded composite fibers’ thermal stability.

#### 2.4.6. CO_2_ Antisolvent

**Figure 16:**
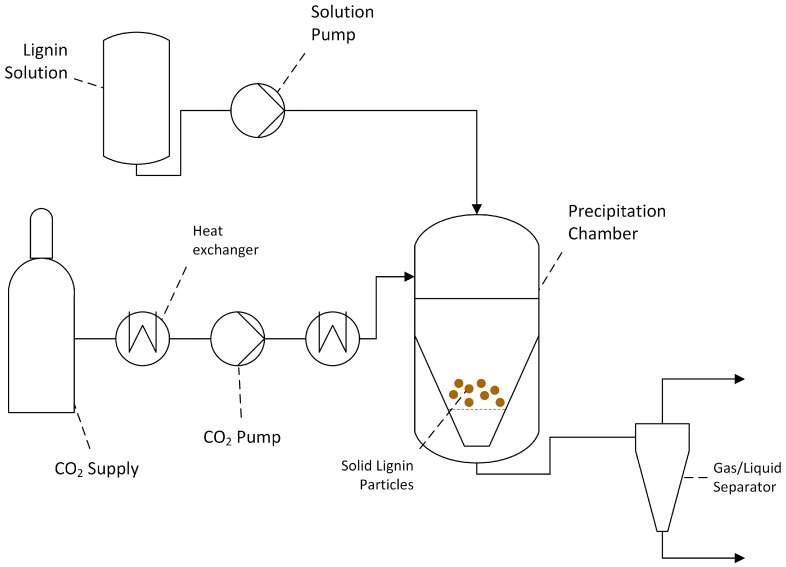
Schematic representation of a CO_2_ precipitation setup.

Over the last two decades, the “compressed/supercritical fluid” based technology used in the production of polymeric nanoparticles has gained considerable attention. Controllability of morphology, size and size distribution is an advantage and especially essential for pharmaceutical and drug delivery applications [130]. CO_2_ is of special interest near its critical conditions (*T*_C_ = 304.3 K and *P*_C_ = 7.4 MPa) due to its tunable physicochemical properties and its abundance, nonflammability, nontoxicity and inexpensiveness [131]. Furthermore, it is a poor solvent for macromolecules such as polymers and therefore an excellent antisolvent [132,133] for precipitation processes where the precipitates can be easily tuned by controlling temperature and pressure [134]. A schematic illustration of a compressed/supercritical fluid setup is shown in Figure 16.

Poplar OS with a purity over 80.5% was used in a supercritical CO_2_ (SC-CO_2_) process by Lu et al. [22]. At first, SC-CO_2_ at 30 MPa and 35 °C, and pure acetone, were injected into the precipitation chamber. After reaching stable reaction conditions, the pure acetone was replaced by 0.5 g/L lignin/acetone solution and precipitated nano-scale particles were taken out from the precipitation chamber’s stainless steel frit. The precipitate was analyzed using DLS, yielding in an average diameter of 144 ± 30 nm and the amorphous structure was determined by X-ray diffraction (XRD). Additionally, the solubility in water was studied and showed 10 and 124 mg/L for non-nanoscale and nanoscale lignin, respectively.

Myint et al. [135] used DMF as a solvent for KL. The procedure and apparatus was similar to the method used by Lu et al. [22]. The aim was to identify the influence of various process parameters (temperature, pressure, solution flow rate and initial solution concentration) on the particles’ product yields, morphology, size, size distribution, surface area and textural properties. The results of these variations in particle size and yield are shown in Table 2. Increasing temperature lead to increased particle diameter, degree of aggregation/coalescence and broader size distributions. Increasing pressure decreased aggregation. Decreasing solution flow rates and increasing lignin concentrations resulted in increased particle diameters and enlargement of the particle size distribution. It is important to mention that this process can achieve yields of almost 90% and surface areas of nearly 92 m^2^/g.

## 3. Comparison

The investigated methods have been compared in terms of solvent consumption, environmental friendliness of solvents, stability of the particles and yields. However, no comprehensive comparison is possible due to a lack of data especially in terms of stability and yield.

Table 3 shows the process parameters of selected methods for which sufficient data is available. The solvent consumption is based on 1 g initial lignin and does not include cleaning and downstream processes. Methods including crosslinking/polymerization and template-based methods are not included due to a considerable variety of different solvents and reacting agents. These methods are discussed separately.

Solvent and antisolvent demands shown in Table 3 are values obtained from laboratory scale experiments and mostly are not optimized. Additionally, a lack of information about the final product yield does not allow for a comprehensive comparison. However, these values deliver an insight into the order of magnitude of solvent demands. Solvent shifting shows the overall highest solvent demand in comparison to other methods shown in Table 3. Nevertheless, precipitation parameters presented by Yearla and Padmasree [41] show promising results for a possible reduction of the solvent demand but yields are strongly dependent on the type of lignin used. Alkali lignin showed a yield of 33% whereas dioxane-extracted lignin showed yields up to 63% applying identical process parameters, indicating that precipitation parameters must be adapted to different types of lignin. Furthermore, Li et al. [52,53,54] could replace the hazardous solvents, dioxane and THF, with more environmentally friendly ethanol to produce hollow particles out of KL. Furthermore, stable particles over a wide range of pH values and NaCl concentrations could be achieved without additional crosslinking agents [34,43].

Methods applying a pH drop have shown to yield solid lignin particles with a greatly reduced antisolvent consumption but lower yield (~10%) [23] compared to solvent-shifting methods yielding up to 90.9% of the initial lignin as nanoparticles [44]. However, due to the lack of published data, this assumption cannot be generalized and depends on the pH-dependent solubility of each specific lignin in the particular solvent. Concerning stability, ethylene glycol and nitric acid as solvent and antisolvent, respectively, for KL yield particles stable in a pH range from 2–10.5 and NaCl concentrations up to 300 mM for 36 month [43].

The methods classified as “other formation methods” show promising results regarding the solvent consumption. In particular, electrospinning allows for high initial lignin loadings of up to 50 wt % [120] and, consequentially, low solvent demand. Supercritical CO_2_ precipitation might show a quite high CO_2_ consumption of up to 41 kg for 1 g of initial lignin [22], but due to the easy recovery and reuse of CO_2_, only the compressor energy demand should be taken into account.

Crosslinking and polymerization methods for the production of lignin particles often include toxic and hazardous reaction agents like epichlorohydrin [21,70,71], hydrogen peroxide [20,87] and toluene diisocyanate [35]. However, these production methods show particular advantages for the encapsulation of active substances due to the simple process of crosslinking in mini and macroemulsions. An important approach for crosslinking without additional chemicals is sonication in miniemulsions which is using the hydroxyl and superoxide radicals generated through sonication for the crosslinking reaction [20]. The different methods of NP formation by crosslinking/polymerization show, especially for the hollow particles, promising applications i.e. in drug delivery or in pickering stabilized emulsions. The size of droplets and therefore, the final size of the capsule in microemulsions can be controlled by adapting the formulation and concentration of surfactants [70,86].

If tailored structures are needed, template-based synthesis can be applied. This method is mostly a multistage process because the template surface must be activated before and removed after the lignin’s deposition. Therefore, this method is worth the expense for specialized applications.

The different methods of NP formation by crosslinking/polymerization show, especially for the hollow particles, promising applications i.e. in drug delivery or in pickering stabilized emulsions. The droplets’ size and therefore the capsule’s final size in microemulsions can be controlled by adapting the formulation and concentration of surfactants.

## 4. Conclusions and Outlook

Different techniques, from simple mechanical treatment to polymerization, were reviewed regarding their application to the formation of lignin nanomaterials. Different shapes of nanomaterials like hollow spheres, open-mouthed hollow spheres, solid spheres, fibers, sheets and tubes can be realized with the techniques available.

Techniques using precipitation methods using a pH drop or a solvent shift are the currently most intensive investigated methods. These methods can form solid as well as hollow nanoparticles but may give rise to some drawbacks regarding industrial applicability. Vast amounts of solvents are needed for purification before precipitation, the precipitation itself and the downstream processing. Some methods even apply environmentally hazardous chemicals such as acetyl bromide to acetylate the lignin before use and broad size distribution might require a separation of desired particle sizes.

Unfortunately, yields of the different methods are rarely reported in literature and differ drastically depending on the type of lignin used. Therefore, increasing yields or the integration in a biorefinery process chain is mandatory and economic improvement should be addressed.

However, lignin structures in nano and microscale will be in many cases only an intermediate. The intermediate and therefore the process must be adapted to the requirements of the final product and the used lignin.

## Figures and Tables

**Table 1:** Morphology results from SEM imaging showing length, diameter, wall thickness and aspect ratio of nanotubes made of lignin from different raw materials and different isolation methods. Adapted with permission from from Ten et al. [101]. Copyright 2014 American Chemical Society.

Raw Material	Isolation Method	Length (μm)	Diameter (nm)	Wall Thickness (nm)	Aspect Ratio
Sugar Cane Bagasse	Phosporic acid	17.0 ± 2.5	200 ± 45	49.5 ± 2.9	85
Sorghum	Klason	15.2 ± 1.4	219.3 ± 71	75.2 ± 16	69
Sorghum (BTx623-bmr6)	Klason	14.3 ± 2.4	223.5 ± 32	59.7 ± 6.7	64
Pine	Klason	15.9 ± 0.9	215.1 ± 33	45.2 ± 2.4	74
Poplar	Klason	16.2 ± 1.0	214.1 ± 39	51.6 ± 4.1	76
Sorghum	Thioglycolic acid	18.6 ± 1.6	219.1 ± 54	44.8 ± 4.3	85
Sorghum (BTx623-bmr6)	Thioglycolic acid	18.0 ± 1.2	203.7 ± 36	51.0 ± 3.6	89
Pine	Thioglycolic acid	17.4 ± 1.4	194.9 ± 20	47.8 ± 3.0	89
Poplar	Thioglycolic acid	17.9 ± 1.4	190.5 ± 99	58.1 ± 7.0	94
Sorghum	NaOH	14.7 ± 1.9	172.4 ± 78	67.9 ± 6.4	85
Sorghum (BTx623-bmr6)	NaOH	15.7 ± 1.5	180.5 ± 74	66.3 ± 7.1	87
Pine	NaOH	10.8 ± 0.7	172.3 ± 28	55.2 ± 3.8	62
Poplar	NaOH	10.9 ± 1.2	178.9 ± 62	69.2 ± 5.6	61

**Table 2:** Mean particle diameter and precipitation yields with variation of Temperature, Pressure, initial lignin solution concentration and solution flowrate. Adapted from Myint et al. [135] with permission of The Royal Society of Chemistry.

T (K)	P (MPa)	Concentration Lignin Solution (wt %)	Flowrate Solution (kg/h)	Mole fraction CO_2_	MPD (nm)	Yield (%)
280.2	15.0	5.3	0.06	0.99	38.0 ± 7.2	77.3
288.2	15.0	5.3	0.06	0.99	51.0 ± 9.8	75.0
298.2	15.0	5.3	0.06	0.98	53.6 ± 11.2	70.0
280.2	7.5	5.3	0.06	0.94	38.0 ± 11.9	75.0
288.2	7.5	5.3	0.06	0.92	47.0 ± 12.8	67.5
298.2	7.5	5.3	0.06	0.90	74.0 ± 17.3	59.0
280.2	15.0	5.3	0.03	0.99	73.0 ± 17.5	51.0
280.2	15.0	10.6	0.06	0.99	54.5 ± 10.7	88.3

**Table 3:** Comparison of investigated methods in terms of lignin type, solvent and antisolvent consumption, yield and stability at different pH and ionic strength values.

Method	Source	Lignin Type ^1^	Solvent ^2^ (L)	Antisolvent ^2^ (L)	Yield (%)	pH Stability	Ionic Strength Stability (mM NaCl)
Solvent shifting (solid particles)	[17]	AL	THF (1)	H_2_O (2.03)	-	<12	-
	[37]	KL	THF (0.1–1)	Dialysis with H_2_O	-	7.4	-
	[34]	KL	THF (0.1–1)	Dialysis with H_2_O	-	4–12	<500
	[38]	OS	THF (0.01–0.1)	H_2_O (0.090–2)	-	-	-
	[43,56]	OS	Acetone (0.2)	H_2_O (1.8)	-	3.5-8	<70
	[40]	EHL/OS	Acetone/H_2_O (0.1–10)	H_2_O (0.4–40)	-	-	-
	[41]	AL/Dioxane	Acetone/ H_2_O (0.1)	H_2_O (0.05)	33–63	-	-
	[44]	EHL	DMSO (0.333–0.488)	Dialysis with H_2_O	41–90.9	4–10	-
Solvent shifting (hollow particles)	[51]	EHL	THF (0.5–2)	H_2_O (2–8)	-	3.5–12	-
	[52]	KL	THF (0.2–2)	H_2_O (1.8–18)	-	-	-
	[54]	KL	Ethanol (0.4)	H_2_O (3.9)	-	-	-
	[53]	KL	Dioxane (0.3)	H_2_O (2.4)	-	-	-
pH shifting (solid particles)	[43,56]	KL	EG (0.2)	0.025M HNO_3_ (0.04–0.12)	-	2–10.5	<300
	[23]	EHL	EG (0.02)	0.25M HCl (0.05)	~10	-	-
	[59]	AL	EG (0.025)	0.1M HCl (0.001) ^3^	-	-	-
	[16]	KL	EG (0.02–0.18)	0.25M HCl (0.03)	-	6–9	-
	[16]	KL	H_2_O/NaOH (2)	0.25M HNO_3_ (0.18)	-	instable	-
	[59]	AL	H_2_O/NaOH (0.011)	0.25M HNO_3_ (to pH 1.9)	-	-	-
Mechanical treatment	[92]	KL	H_2_O (0.2)	-	-	-	-
	[36]	AL	H_2_O (1.4)	-	-	-	-
Ice segregation	[98]	AL	DMSO (0.2–5)	Dialysis with H_2_O	-	10.5	-
	[97]	AL	H_2_O/NaOH (3.3–10)	-	-	-	-
Aerosol processing	[114]	AL	H_2_O (0.05–0.2)	-	>60	-	-
		KL/OS	DMF (0.05–0.2)	-	>60	-	-
Electro-spinning	[124]	OS	Ethanol (0.0013)	-	-	-	-
	[120]	KL/OS/PL/LS	DMF (0.001–0.01)H_2_O (0.001–0.01)	-	-	-	-
	[129]	KL	PVA/H_2_O (0.002)	-	-	-	-
CO_2_ precipitation	[135]	KL	DMF (0.008–0.018)	CO_2_ (0.47–1.89 kg)	51–88.3	-	-
	[22]	OS	Acetone (2)	CO_2_ (41 kg)	-	-	-

^1^ Kraft lignin (KL), Organosolv lignin (OS), Enzymatic hydrolysis lignin (EHL), Lignosulphonate (LS), Pyrolytic Lignin (PL); ^2^ based on 1 g initial lignin; ^3^ additional crosslinking with 30 mL 0.4 wt % glutaraldehyde.

## Abbreviations

ACL	Acetylated lignin
AL	Alkali lignin
APTES	(3-aminopropyl)-triethoxysilane
CL	Cationic lignin
DHP	Dehydrogenation polymer
DLS	Dynamic Light Scattering
DMF	Dimethylformamide
DMSO	Dimethyl sulfoxide
EG	Ethylene glycol
EHL	Enzymatic hydrolysis lignin
ISISA	Ice- segregation-induced self-assembly
KL	Kraft lignin
MMT	Montmorillonite
NP	Nanoparticle
OS	Organosolv lignin
PDAC	Poly(diallyldimethylammonium chloride)
PEGDEG	Poly(ethylene glycol) diglycidyl ether
PEO	Poly(ethylene oxide)
PL	Pyrolytic lignin
RAFT	Reversible addition-fragmentation chain transfer
SEM	Scanning electron microscopy
SLI	Scattered light intensity
TEM	Transmission electron microscopy
THF	Tetrahydrofuran
XRD	X-ray diffraction
